# 4-{Phen­yl[4-(6-phenyl-2,2′-bipyridin-4-yl)phen­yl]amino}­benzaldehyde

**DOI:** 10.1107/S1600536814013361

**Published:** 2014-07-02

**Authors:** Yu-yang Zhang, Jian-Ting Pan, Jian-Yan Huang

**Affiliations:** a Department of Chemistry, Anhui University, Hefei 230039, People’s Republic of China; b Key Laboratory of Functional Inorganic Materials Chemistry, Hefei 230039, People’s Republic of China

## Abstract

The title mol­ecule, C_35_H_25_N_3_O, is a tri­phenyl­amine derivative with the 4-position substituted by an aldehyde group, and the 4′-position substituted by a 6-phenyl-2,2′-bi­pyridine group. The whole mol­ecule is non-planar and the dihedral angle between the core benzene and pyridine rings is 36.96 (5)°. The dihedral angle between the phenyl and benzaldehyde groups bonded to the amine N atom is 70.86 (5)°.

## Related literature   

For the application of the title compound and related mol­ecules in OLED devices, see: Neve *et al.* (2002 ▶); Lu *et al.* (2004 ▶); Ye *et al.* (2010 ▶). For a related mol­ecule and its application in synthesis, see: Shen *et al.* (2012 ▶).
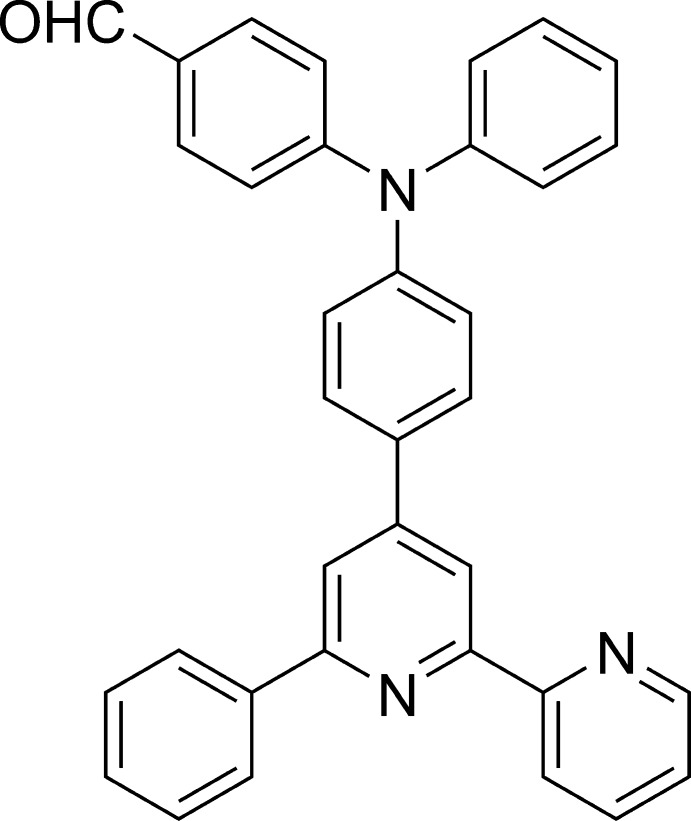


## Experimental   

### 

#### Crystal data  

C_35_H_25_N_3_O
*M* *_r_* = 503.58
Monoclinic, 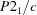

*a* = 14.4204 (9) Å
*b* = 10.0329 (6) Å
*c* = 18.4597 (11) Å
β = 101.423 (1)°
*V* = 2617.8 (3) Å^3^
*Z* = 4
Mo *K*α radiation
μ = 0.08 mm^−1^
*T* = 296 K
0.30 × 0.20 × 0.20 mm

#### Data collection  

Bruker APEXII CCD diffractometer
13094 measured reflections
4580 independent reflections
3379 reflections with *I* > 2σ(*I*)
*R*_int_ = 0.024

#### Refinement  

*R*[*F* ^2^ > 2σ(*F* ^2^)] = 0.039
*wR*(*F* ^2^) = 0.097
*S* = 0.99
4580 reflections
352 parameters
H-atom parameters constrained
Δρ_max_ = 0.12 e Å^−3^
Δρ_min_ = −0.15 e Å^−3^

### 

Data collection: *SMART* (Bruker, 2007 ▶); cell refinement: *SAINT* (Bruker, 2007 ▶); data reduction: *SAINT*; program(s) used to solve structure: *SHELXS97* (Sheldrick, 2008 ▶); program(s) used to refine structure: *SHELXL97* (Sheldrick, 2008 ▶); molecular graphics: *SHELXTL* (Sheldrick, 2008 ▶); software used to prepare material for publication: *SHELXTL*.

## Supplementary Material

Crystal structure: contains datablock(s) I, Global. DOI: 10.1107/S1600536814013361/bh2499sup1.cif

Structure factors: contains datablock(s) I. DOI: 10.1107/S1600536814013361/bh2499Isup2.hkl

Supporting information file. DOI: 10.1107/S1600536814013361/bh2499Isup3.cml

CCDC reference: 965698

Additional supporting information:  crystallographic information; 3D view; checkCIF report

## Figures and Tables

**Fig. 1.**
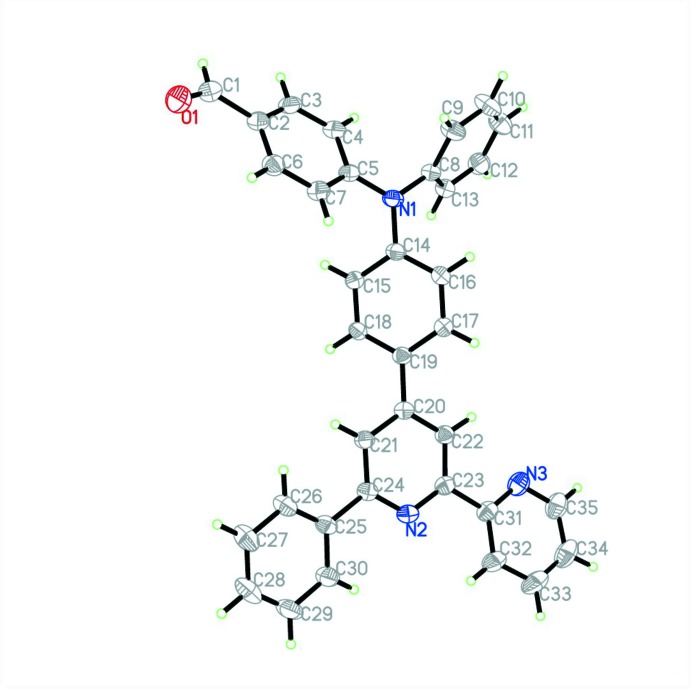
The molecular structure of the title compound.

## supplementary crystallographic information

### S1. Comment

The title compound (Fig. 1) includes a triphenylamine group and a C^N^N (HC^N^N = 6-aryl-2,2'-bipyridine) moiety. The triphenylamine group has an extended conjugated system, and is usually used in organic light-emitting diodes (OLED), due to its high holes mobility (Ye *et al.*, 2010). The C^N^N moiety is a better donor than terpyridine and has a better π-acceptor ability than the C^N^C moiety (HC^N^CH = 2, 6-diphenylpyridine) (Lu *et al.*, 2004). The title compound can be used as an intermediate for 6-aryl-2,2'-bipyridine metal complexes (Shen *et al.*, 2012) and may find applications in light-emitting devices and dye-sensitized devices (Neve *et al.*, 2002).

The bond lengths around the amine N atom, N1—C5, N1—C8 and N1—C14, differ from each other, which are 1.4079 (19), 1.4285 (18) and 1.4256 (18) Å, respectively. The bond distance of C23—C31 is almost equal to the bond distance of C24—C25, but the dihedral angles of the phenyl group and pyridine moiety is slightly different from that of pyridine moiety and the terminal pyridine ring, which are 10.45 and 14.49°, respectively. The central core of rings is also twisted, with the torsion angle C17—C19—C20—C21 being 142.01°.

### S2. Experimental

4-(Diphenylamino)benzaldehyde (1.00 g), acetophenone (0.88 g), and NaOH (0.22 g) were dissolved in 10 ml of ethanol and the mixture was refluxed for about 12 h. The precipitate was filtered, purified by recrystallization from ethanol, yielding 1.21 g of yellow solid, (*E*)-3-[4-(diphenylamino)phenyl]-1-phenylprop-2-en-1-one (D1). Yield: 88%. D1 (1.00 g), 1-(pyridin-2-yl)ethanone (0.32 g), and NaOH (0.13 g) were crushed together with a pestle and mortar at room temperature for 1 h. The mixture was purified by recrystallization from ethanol, affording 1.2 g of solid, 3-[4-(diphenylamino)phenyl]-1-phenyl-5-(pyridin-2-yl)pentane-1,5-dione (D2). Yield: 91%. D2 (1.00 g) and ammonium acetate (4.66 g) were dissolved in 20 ml of ethanol and refluxed for 24 h. The precipitate was filtered, purified by recrystallization from a mixture of dichloromethane and ethanol, to give 0.85 g of a yellow solid, *N*,*N*-diphenyl-4-(6-phenyl-2,2'-bipyridin-4-yl)aniline (D3). Yield: 89%. The title compound was obtained through the Vilsmeier-Haack reaction of D3 (0.85 g). The precipitate was purified by flash chromatography on silica gel using petroleum/ethyl acetate (8:1) as eluent, affording 0.62 g of a yellow solid. Yield: 69%. ^1^H NMR (400 MHz, CDCl_3_) 7.13 (d, *J* = 8.0 Hz, 2H), 7.22 (d, *J* = 8.0 Hz, 3H), 7.29 (t, 2H), 7.37 (m, 3H), 7.46 (t, 1H), 7.53 (t, 2H), 7.73 (t, 2H), 7.79 (d, *J* = 8.0 Hz, 2H), 7.87 (t, 1H), 7.97 (s, 1H), 8.21 (d, *J* = 7.6 Hz, 2H), 8.63 (s, 1H), 8.70 (t, 2H), 9.85 ppm (s, 1H). ^13^C NMR (100 MHz) 117.18, 118.04, 120.38, 121.54, 123.90, 125.47, 125.83, 126.49, 127.09, 128.54, 128.79, 129.16, 129.18, 129.92, 131.38, 134.79, 136.94, 139.44, 146.00, 147.12, 149.08, 149.33, 152.96, 156.28, 156.34, 157.23, 190.54 ppm.

### S3. Refinement

All H atoms were placed in geometrically idealized positions and constrained to ride on their parent atoms, with C—H = 0.93 Å and *U*_iso_(H) = 1.2 *U*_eq_(carrier C).

### Crystal data

**Table:**

C_35_H_25_N_3_O	*F*(000) = 1056
*M**_r_* = 503.58	*D*_x_ = 1.278 Mg m^−^^3^
Monoclinic, *P*2_1_/*c*	Mo *K*α radiation, λ = 0.71073 Å
Hall symbol: -P 2ybc	Cell parameters from 3436 reflections
*a* = 14.4204 (9) Å	θ = 2.3–27.3°
*b* = 10.0329 (6) Å	µ = 0.08 mm^−^^1^
*c* = 18.4597 (11) Å	*T* = 296 K
β = 101.423 (1)°	Needle, yellow
*V* = 2617.8 (3) Å^3^	0.30 × 0.20 × 0.20 mm
*Z* = 4	

### Data collection

**Table:**

Bruker APEXII CCD diffractometer	3379 reflections with *I* > 2σ(*I*)
Radiation source: fine-focus sealed tube	*R*_int_ = 0.024
Graphite monochromator	θ_max_ = 25.0°, θ_min_ = 2.3°
φ and ω scans	*h* = −16→17
13094 measured reflections	*k* = −11→11
4580 independent reflections	*l* = −19→21

### Refinement

**Table:**

Refinement on *F*^2^	Primary atom site location: structure-invariant direct methods
Least-squares matrix: full	Secondary atom site location: difference Fourier map
*R*[*F*^2^ > 2σ(*F*^2^)] = 0.039	Hydrogen site location: inferred from neighbouring sites
*wR*(*F*^2^) = 0.097	H-atom parameters constrained
*S* = 0.99	*w* = 1/[σ^2^(*F*_o_^2^) + (0.0368*P*)^2^ + 0.5245*P*] where *P* = (*F*_o_^2^ + 2*F*_c_^2^)/3
4580 reflections	(Δ/σ)_max_ = 0.001
352 parameters	Δρ_max_ = 0.12 e Å^−^^3^
0 restraints	Δρ_min_ = −0.15 e Å^−^^3^
0 constraints	

### Fractional atomic coordinates and isotropic or equivalent isotropic displacement parameters (Å^2^)

**Table:**

	*x*	*y*	*z*	*U*_iso_*/*U*_eq_	
C1	0.37069 (18)	0.99664 (19)	0.85338 (11)	0.0791 (6)	
H1	0.4274	1.0401	0.8523	0.095*	
C2	0.37390 (13)	0.88662 (15)	0.90600 (9)	0.0578 (4)	
C3	0.45768 (13)	0.85771 (16)	0.95466 (10)	0.0594 (4)	
H3	0.5119	0.9061	0.9519	0.071*	
C4	0.46236 (11)	0.75857 (15)	1.00714 (9)	0.0535 (4)	
H4	0.5189	0.7428	1.0402	0.064*	
C5	0.38289 (11)	0.68211 (14)	1.01074 (8)	0.0464 (4)	
C6	0.29420 (12)	0.81128 (16)	0.91074 (9)	0.0574 (4)	
H6	0.2372	0.8295	0.8788	0.069*	
C7	0.29833 (11)	0.71064 (16)	0.96165 (9)	0.0525 (4)	
H7	0.2444	0.6610	0.9635	0.063*	
C8	0.45874 (11)	0.58033 (15)	1.12868 (8)	0.0480 (4)	
C9	0.46558 (13)	0.68847 (18)	1.17592 (10)	0.0677 (5)	
H9	0.4232	0.7590	1.1654	0.081*	
C10	0.53571 (16)	0.6910 (2)	1.23866 (11)	0.0862 (6)	
H10	0.5412	0.7646	1.2699	0.103*	
C11	0.59739 (15)	0.5868 (2)	1.25570 (11)	0.0813 (6)	
H11	0.6440	0.5892	1.2984	0.098*	
C12	0.59008 (12)	0.4788 (2)	1.20945 (10)	0.0659 (5)	
H12	0.6318	0.4077	1.2208	0.079*	
C13	0.52092 (11)	0.47552 (16)	1.14605 (9)	0.0532 (4)	
H13	0.5162	0.4020	1.1148	0.064*	
C14	0.33521 (10)	0.45819 (14)	1.04475 (8)	0.0444 (4)	
C15	0.32065 (10)	0.40549 (14)	0.97396 (8)	0.0455 (4)	
H15	0.3448	0.4494	0.9373	0.055*	
C16	0.30076 (11)	0.38972 (15)	1.09888 (8)	0.0498 (4)	
H16	0.3111	0.4228	1.1469	0.060*	
C17	0.25108 (11)	0.27239 (15)	1.08197 (8)	0.0496 (4)	
H17	0.2285	0.2275	1.1190	0.059*	
C18	0.27064 (10)	0.28839 (14)	0.95742 (8)	0.0448 (4)	
H18	0.2612	0.2548	0.9096	0.054*	
C19	0.23406 (10)	0.21983 (14)	1.01084 (8)	0.0428 (3)	
C20	0.17668 (10)	0.09761 (14)	0.99163 (8)	0.0440 (4)	
C21	0.11550 (10)	0.08717 (15)	0.92369 (8)	0.0463 (4)	
H21	0.1127	0.1554	0.8892	0.056*	
C22	0.17932 (11)	−0.00827 (15)	1.04009 (9)	0.0482 (4)	
H22	0.2190	−0.0054	1.0864	0.058*	
C23	0.12231 (10)	−0.11869 (15)	1.01913 (9)	0.0471 (4)	
C24	0.05842 (10)	−0.02439 (15)	0.90672 (8)	0.0458 (4)	
C25	−0.01267 (10)	−0.03392 (16)	0.83667 (9)	0.0491 (4)	
C26	−0.03301 (12)	0.07457 (17)	0.79072 (9)	0.0605 (5)	
H26	0.0004	0.1535	0.8024	0.073*	
C27	−0.10196 (14)	0.0679 (2)	0.72791 (10)	0.0768 (6)	
H27	−0.1148	0.1421	0.6974	0.092*	
C28	−0.15196 (14)	−0.0471 (2)	0.70983 (11)	0.0812 (6)	
H28	−0.1993	−0.0507	0.6676	0.097*	
C29	−0.13200 (14)	−0.1569 (2)	0.75411 (11)	0.0803 (6)	
H29	−0.1651	−0.2358	0.7416	0.096*	
C30	−0.06295 (12)	−0.15054 (19)	0.81716 (10)	0.0655 (5)	
H30	−0.0498	−0.2255	0.8471	0.079*	
C31	0.12354 (12)	−0.23332 (15)	1.07047 (9)	0.0525 (4)	
C32	0.05216 (13)	−0.32814 (17)	1.05889 (11)	0.0671 (5)	
H32	0.0041	−0.3243	1.0171	0.081*	
C33	0.05354 (17)	−0.42849 (19)	1.11039 (14)	0.0840 (6)	
H33	0.0061	−0.4927	1.1040	0.101*	
C34	0.12583 (18)	−0.4321 (2)	1.17110 (13)	0.0866 (7)	
H34	0.1278	−0.4980	1.2068	0.104*	
C35	0.19494 (16)	−0.3370 (2)	1.17810 (11)	0.0779 (6)	
H35	0.2443	−0.3413	1.2190	0.093*	
N1	0.38793 (9)	0.57802 (12)	1.06252 (7)	0.0509 (3)	
N2	0.06237 (9)	−0.12729 (12)	0.95393 (7)	0.0495 (3)	
N3	0.19577 (11)	−0.23785 (14)	1.12957 (8)	0.0671 (4)	
O1	0.30133 (13)	1.03588 (14)	0.81138 (8)	0.1031 (5)	

### Atomic displacement parameters (Å^2^)

**Table:**

	*U*^11^	*U*^22^	*U*^33^	*U*^12^	*U*^13^	*U*^23^
C1	0.1167 (18)	0.0504 (12)	0.0663 (13)	−0.0151 (11)	0.0088 (12)	−0.0004 (9)
C2	0.0786 (12)	0.0372 (9)	0.0575 (10)	−0.0051 (8)	0.0130 (9)	−0.0056 (8)
C3	0.0693 (11)	0.0409 (9)	0.0693 (11)	−0.0146 (8)	0.0165 (9)	−0.0076 (8)
C4	0.0525 (9)	0.0423 (9)	0.0634 (10)	−0.0052 (7)	0.0057 (8)	−0.0071 (8)
C5	0.0516 (9)	0.0341 (8)	0.0534 (9)	−0.0024 (7)	0.0100 (7)	−0.0070 (7)
C6	0.0640 (11)	0.0464 (10)	0.0584 (10)	0.0044 (8)	0.0040 (8)	−0.0031 (8)
C7	0.0514 (9)	0.0439 (9)	0.0613 (10)	−0.0018 (7)	0.0090 (8)	−0.0019 (8)
C8	0.0517 (9)	0.0446 (9)	0.0463 (9)	−0.0109 (7)	0.0064 (7)	−0.0059 (7)
C9	0.0750 (12)	0.0563 (11)	0.0679 (12)	−0.0007 (9)	0.0044 (10)	−0.0186 (9)
C10	0.0982 (16)	0.0834 (15)	0.0690 (13)	−0.0116 (13)	−0.0029 (12)	−0.0342 (11)
C11	0.0761 (14)	0.0958 (16)	0.0616 (12)	−0.0156 (12)	−0.0117 (10)	−0.0108 (12)
C12	0.0583 (11)	0.0707 (12)	0.0644 (11)	−0.0019 (9)	0.0018 (9)	0.0059 (10)
C13	0.0587 (10)	0.0493 (9)	0.0509 (9)	−0.0054 (8)	0.0094 (8)	−0.0040 (7)
C14	0.0439 (8)	0.0382 (8)	0.0483 (9)	−0.0039 (6)	0.0027 (7)	−0.0028 (7)
C15	0.0477 (9)	0.0428 (9)	0.0448 (9)	−0.0048 (7)	0.0067 (7)	0.0019 (7)
C16	0.0557 (10)	0.0498 (9)	0.0427 (9)	−0.0058 (8)	0.0065 (7)	−0.0052 (7)
C17	0.0529 (9)	0.0497 (9)	0.0456 (9)	−0.0076 (7)	0.0087 (7)	0.0014 (7)
C18	0.0485 (9)	0.0431 (9)	0.0399 (8)	−0.0037 (7)	0.0016 (7)	−0.0024 (7)
C19	0.0402 (8)	0.0389 (8)	0.0461 (8)	−0.0013 (6)	0.0011 (6)	0.0017 (7)
C20	0.0406 (8)	0.0402 (8)	0.0503 (9)	−0.0017 (6)	0.0070 (7)	−0.0021 (7)
C21	0.0452 (9)	0.0412 (8)	0.0507 (9)	−0.0041 (7)	0.0052 (7)	0.0012 (7)
C22	0.0485 (9)	0.0434 (9)	0.0504 (9)	−0.0012 (7)	0.0043 (7)	0.0009 (7)
C23	0.0468 (9)	0.0398 (9)	0.0563 (10)	0.0013 (7)	0.0141 (8)	−0.0008 (7)
C24	0.0395 (8)	0.0447 (9)	0.0530 (9)	−0.0002 (7)	0.0088 (7)	−0.0062 (7)
C25	0.0396 (8)	0.0544 (10)	0.0531 (9)	−0.0021 (7)	0.0087 (7)	−0.0129 (8)
C26	0.0576 (10)	0.0561 (11)	0.0597 (11)	−0.0005 (8)	−0.0078 (8)	−0.0100 (9)
C27	0.0779 (13)	0.0788 (14)	0.0638 (12)	0.0131 (11)	−0.0100 (10)	−0.0118 (10)
C28	0.0628 (12)	0.1089 (18)	0.0632 (13)	−0.0024 (12)	−0.0087 (10)	−0.0298 (13)
C29	0.0735 (13)	0.0929 (16)	0.0706 (13)	−0.0309 (12)	0.0050 (11)	−0.0278 (12)
C30	0.0649 (11)	0.0687 (12)	0.0622 (11)	−0.0189 (9)	0.0105 (9)	−0.0156 (9)
C31	0.0577 (10)	0.0404 (9)	0.0624 (10)	0.0032 (7)	0.0196 (9)	0.0016 (8)
C32	0.0744 (12)	0.0468 (10)	0.0840 (13)	−0.0080 (9)	0.0253 (10)	0.0021 (9)
C33	0.0977 (16)	0.0487 (12)	0.1151 (18)	−0.0098 (11)	0.0440 (15)	0.0100 (12)
C34	0.1124 (18)	0.0597 (13)	0.0973 (17)	0.0124 (13)	0.0443 (15)	0.0286 (12)
C35	0.0916 (15)	0.0655 (13)	0.0790 (14)	0.0144 (11)	0.0229 (11)	0.0226 (11)
N1	0.0563 (8)	0.0399 (7)	0.0519 (8)	−0.0098 (6)	−0.0004 (6)	−0.0030 (6)
N2	0.0468 (7)	0.0425 (7)	0.0593 (8)	−0.0037 (6)	0.0106 (6)	−0.0037 (6)
N3	0.0734 (10)	0.0582 (9)	0.0706 (10)	0.0078 (7)	0.0166 (8)	0.0156 (8)
O1	0.1452 (15)	0.0692 (10)	0.0818 (10)	−0.0054 (9)	−0.0088 (10)	0.0165 (8)

### Geometric parameters (Å, º)

**Table:**

C1—O1	1.204 (2)	C18—C19	1.389 (2)
C1—C2	1.465 (2)	C18—H18	0.9300
C1—H1	0.9300	C19—C20	1.4826 (19)
C2—C3	1.386 (2)	C20—C22	1.384 (2)
C2—C6	1.393 (2)	C20—C21	1.3870 (19)
C3—C4	1.381 (2)	C21—C24	1.388 (2)
C3—H3	0.9300	C21—H21	0.9300
C4—C5	1.391 (2)	C22—C23	1.388 (2)
C4—H4	0.9300	C22—H22	0.9300
C5—C7	1.397 (2)	C23—N2	1.3384 (19)
C5—N1	1.4079 (19)	C23—C31	1.488 (2)
C6—C7	1.373 (2)	C24—N2	1.3449 (19)
C6—H6	0.9300	C24—C25	1.486 (2)
C7—H7	0.9300	C25—C26	1.375 (2)
C8—C13	1.378 (2)	C25—C30	1.386 (2)
C8—C9	1.383 (2)	C26—C27	1.371 (2)
C8—N1	1.4285 (18)	C26—H26	0.9300
C9—C10	1.379 (3)	C27—C28	1.367 (3)
C9—H9	0.9300	C27—H27	0.9300
C10—C11	1.368 (3)	C28—C29	1.368 (3)
C10—H10	0.9300	C28—H28	0.9300
C11—C12	1.370 (3)	C29—C30	1.375 (2)
C11—H11	0.9300	C29—H29	0.9300
C12—C13	1.379 (2)	C30—H30	0.9300
C12—H12	0.9300	C31—N3	1.352 (2)
C13—H13	0.9300	C31—C32	1.387 (2)
C14—C15	1.387 (2)	C32—C33	1.382 (3)
C14—C16	1.383 (2)	C32—H32	0.9300
C14—N1	1.4256 (18)	C33—C34	1.371 (3)
C15—C18	1.381 (2)	C33—H33	0.9300
C15—H15	0.9300	C34—C35	1.368 (3)
C16—C17	1.381 (2)	C34—H34	0.9300
C16—H16	0.9300	C35—N3	1.340 (2)
C17—C19	1.391 (2)	C35—H35	0.9300
C17—H17	0.9300		
			
O1—C1—C2	125.9 (2)	C18—C19—C20	120.80 (13)
O1—C1—H1	117.0	C17—C19—C20	121.68 (13)
C2—C1—H1	117.0	C22—C20—C21	117.23 (14)
C3—C2—C6	118.04 (16)	C22—C20—C19	122.51 (13)
C3—C2—C1	119.76 (17)	C21—C20—C19	120.25 (13)
C6—C2—C1	122.17 (18)	C20—C21—C24	120.50 (14)
C2—C3—C4	121.38 (16)	C20—C21—H21	119.7
C2—C3—H3	119.3	C24—C21—H21	119.7
C4—C3—H3	119.3	C20—C22—C23	119.56 (14)
C3—C4—C5	120.28 (15)	C20—C22—H22	120.2
C3—C4—H4	119.9	C23—C22—H22	120.2
C5—C4—H4	119.9	N2—C23—C22	122.92 (14)
C4—C5—C7	118.52 (15)	N2—C23—C31	116.55 (14)
C4—C5—N1	120.60 (14)	C22—C23—C31	120.50 (14)
C7—C5—N1	120.88 (14)	N2—C24—C21	121.71 (14)
C7—C6—C2	121.19 (16)	N2—C24—C25	116.61 (13)
C7—C6—H6	119.4	C21—C24—C25	121.64 (14)
C2—C6—H6	119.4	C26—C25—C30	118.11 (16)
C6—C7—C5	120.57 (15)	C26—C25—C24	120.79 (14)
C6—C7—H7	119.7	C30—C25—C24	121.05 (16)
C5—C7—H7	119.7	C25—C26—C27	120.87 (17)
C13—C8—C9	119.34 (15)	C25—C26—H26	119.6
C13—C8—N1	120.57 (14)	C27—C26—H26	119.6
C9—C8—N1	120.09 (15)	C26—C27—C28	120.4 (2)
C10—C9—C8	119.52 (18)	C26—C27—H27	119.8
C10—C9—H9	120.2	C28—C27—H27	119.8
C8—C9—H9	120.2	C29—C28—C27	119.71 (18)
C11—C10—C9	120.97 (18)	C29—C28—H28	120.1
C11—C10—H10	119.5	C27—C28—H28	120.1
C9—C10—H10	119.5	C28—C29—C30	119.99 (19)
C10—C11—C12	119.63 (18)	C28—C29—H29	120.0
C10—C11—H11	120.2	C30—C29—H29	120.0
C12—C11—H11	120.2	C29—C30—C25	120.86 (19)
C11—C12—C13	120.07 (18)	C29—C30—H30	119.6
C11—C12—H12	120.0	C25—C30—H30	119.6
C13—C12—H12	120.0	N3—C31—C32	122.08 (16)
C8—C13—C12	120.46 (16)	N3—C31—C23	116.75 (14)
C8—C13—H13	119.8	C32—C31—C23	121.14 (16)
C12—C13—H13	119.8	C33—C32—C31	118.99 (19)
C15—C14—C16	118.84 (13)	C33—C32—H32	120.5
C15—C14—N1	120.85 (13)	C31—C32—H32	120.5
C16—C14—N1	120.26 (13)	C34—C33—C32	119.1 (2)
C14—C15—C18	120.52 (14)	C34—C33—H33	120.4
C14—C15—H15	119.7	C32—C33—H33	120.4
C18—C15—H15	119.7	C35—C34—C33	118.71 (19)
C17—C16—C14	120.29 (14)	C35—C34—H34	120.6
C17—C16—H16	119.9	C33—C34—H34	120.6
C14—C16—H16	119.9	N3—C35—C34	123.9 (2)
C16—C17—C19	121.53 (14)	N3—C35—H35	118.1
C16—C17—H17	119.2	C34—C35—H35	118.1
C19—C17—H17	119.2	C5—N1—C14	121.05 (12)
C15—C18—C19	121.30 (14)	C5—N1—C8	120.03 (12)
C15—C18—H18	119.4	C14—N1—C8	117.96 (12)
C19—C18—H18	119.4	C23—N2—C24	118.04 (13)
C18—C19—C17	117.49 (13)	C35—N3—C31	117.20 (17)
